# Fructose metabolism is unregulated in cancers and placentae

**DOI:** 10.3389/ebm.2024.10200

**Published:** 2024-10-28

**Authors:** Fuller W. Bazer, Guoyao Wu, Gregory A. Johnson

**Affiliations:** ^1^ Department of Animal Science, Texas A&M University, College Station, TX, United States; ^2^ Department of Veterinary Integrative Biosciences, Texas A&M University, College Station, TX, United States

**Keywords:** glucose, fructose, lactate, pregnancy, tumor biology, placenta

## Abstract

Fructose and lactate are present in high concentrations in uterine luminal fluid, fetal fluids and fetal blood of ungulates and cetaceans, but their roles have been ignored and they have been considered waste products of pregnancy. This review provides evidence for key roles of both fructose and lactate in support of key metabolic pathways required for growth and development of fetal-placental tissues, implantation and placentation. The uterus and placenta of ungulates convert glucose to fructose via the polyol pathway. Fructose is sequestered within the uterus and cannot be transported back into the maternal circulation. Fructose is phosphorylated by ketohexokinase to fructose-1-PO4 (F1P) by that is metabolized via the fructolysis pathway to yield dihydoxyacetone phosphate and glyceraldehyde-3-PO4 that are downstream of phosphofructokinase. Thus, there is no inhibition of the fructolysis pathway by low pH, citrate or ATP which allows F1P to continuously generate substrates for the pentose cycle, hexosamine biosynthesis pathway, one-carbon metabolism and tricarboxylic acid cycle, as well as lactate. Lactate sustains the activity of hypoxia-inducible factor alpha and its downstream targets such as vascular endothelial growth factor to increase utero-placental blood flow critical to growth and development of the fetal-placental tissues and a successful outcome of pregnancy. Pregnancy has been referred to as a controlled cancer and this review addresses similarities regarding metabolic aspects of tumors and the placenta.

## Introduction

Proliferating cells, such as cancer cells and activated lymphocytes, are metabolically different from nonproliferating cells as they are programmed to utilize either anaerobic or aerobic glycolysis, the Warburg effect, depending on the availability of oxygen [1]. Glucose and fructose are hexose sugars that provide glycolytic intermediates for further metabolism via the pentose cycle, tricarboxylic acid cycle (TCA), one-carbon metabolism and hexosamine biosynthesis pathway. Glycolysis is a physiological response of tissues to hypoxia, a low oxygen environment, with cells of tumors taking up glucose and producing intermediate products of glycolysis (including fructose-6-phosphate, F6P) and significant amounts of lactate. By switching from oxidative phosphorylation to glycolysis, cells rapidly generate ATP as compared with oxidation of glucose and activation of the TCA cycle. Further, glycolytic intermediates are substrates for other metabolic pathways required to meet demands for proliferation, migration, and differentiation of cells [2–5].

**FIGURE 1:**
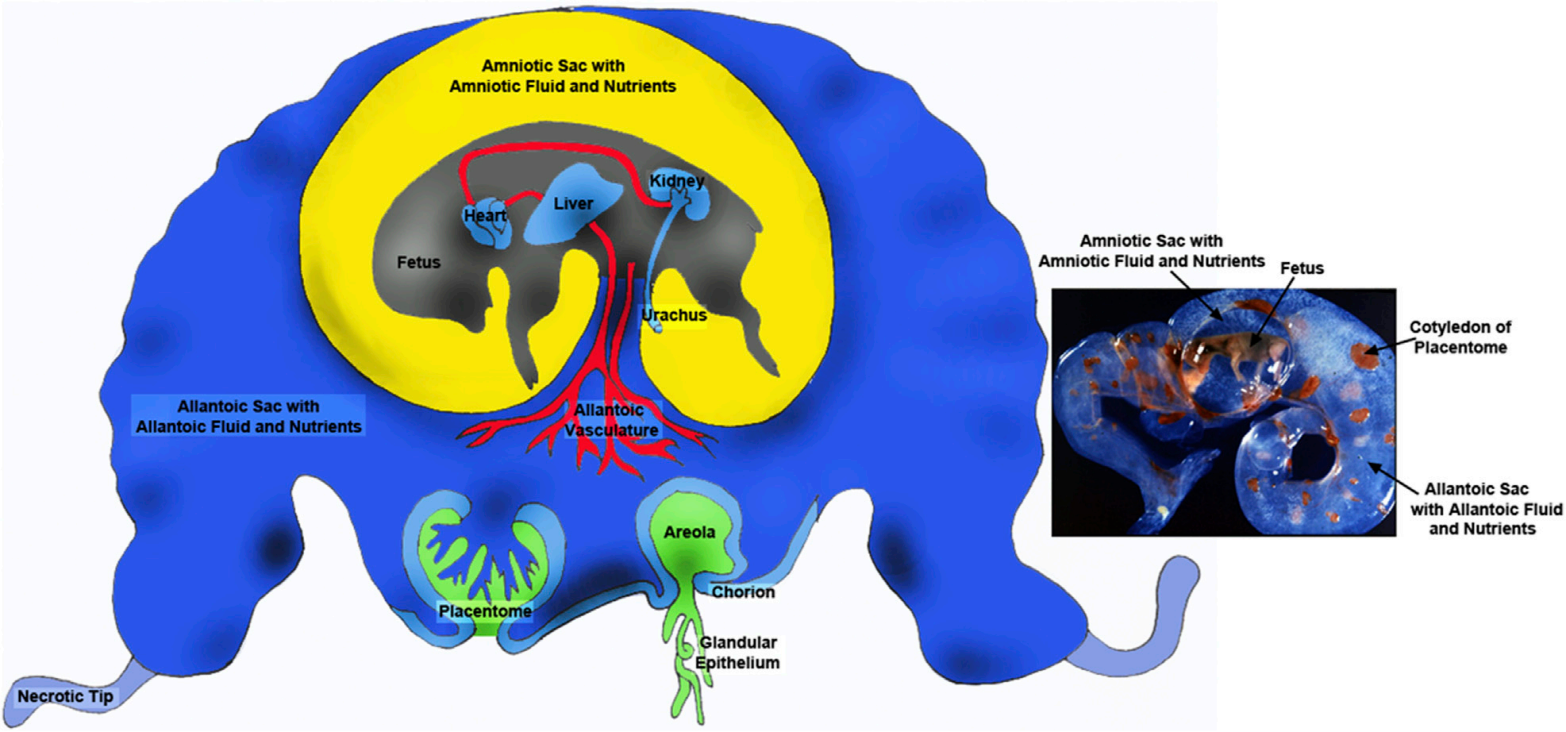
Placentae of ungulates include the amnion, allantois and chorion with the latter two membranes forming the chorioallantois. The majority of blood flow is via placentomes composed on the caruncle on the maternal side (green portion) and the cotyledon on the fetal side (blue). There are also areolae associated with the openings of uterine glands that form a “pocket” into which secretions are delivered and transported across the chorioallantois intothe fetal-placental circulation. The amnion is filled with amniotic fluid that is basically isosmotic to fetal serum and contains nutrients such as glucose and fructose. The allantoic sac is filled with allantoic fluid of which changes in are dynamic manner throughout gestation. Although allantoic fluid is hypoosmotic with respect to fetal fluids it is enriched in nutrients transported for support of fetal-placental tissues or cleared through the kidney into the bladder for delivery back into the allantoic sac via the urachus. Thus, allantoic fluid is a nutrient reservoir for the developing conceptus.

This review focuses on the metabolism of glucose and fructose, two molecules present in great abundances in conceptuses (embryo and extra-embryonic membranes; fetus and placenta) of ungulates and cetaceans throughout pregnancy [see [6]; Figure 1]. Fructose and lactate have been considered metabolic wastes, so their functional roles in conceptus development have not been established. The following sections of this review evidence for important roles of fructose and lactate in conceptus development. Thus, the focus of this review is on contributions of glucose and fructose to the major metabolic pathways required for development of conceptuses under oxygenated and low oxygen conditions, expression of enzymes for production and metabolism of fructose from glucose, and characterization of lactate synthesis and transport throughout pregnancy.

Another focus of this review is the role of fructose in uncontrolled growth of cancers, as Burton et al. [7] reported similarities between metabolism in placentae and in malignant tumors while acknowledging that growth of the placenta is regulated. They note that the availability of oxygen controls placental development and tumor behavior as both develop in a low oxygen environment, and both need to stimulate vascular development to deliver nutrients and oxygen sufficient to support high rates of cell proliferation. Thus, glycolysis is common in support of growth and development of both placentae and tumors. Glycolysis, rather than oxidative phosphorylation, has the advantage of maintaining carbon skeletons for the synthesis of nucleotides, cell membranes and organelles, as well as protecting the conceptus from adverse effects of free radicals.

## Fructose metabolism and cancer (see Krause and Wegner)

The link between cancer and altered glucose metabolism was discovered by Otto Warburg over 100 years ago. A connection between cancer and fructose metabolism was also discovered as fructose, like glucose, affects growth, proliferation, and survival of cancer cells [8, 9]. Cancer cells express solute carrier family member 5 (SLC2A5), the fructose transporter, and upregulation of SLC2A5 is indicative of a poor prognosis for cancer patients that experienced an increase in cancer cell proliferation, colony growth, and metastasis [10]. There is over-expression of SLC2A5 for glioblastoma, colon, liver, lung, breast, and prostate cancers [11, 12].

The polyol pathway involves two steps for conversion of glucose to fructose [13–15]. Glucose is reduced to sorbitol by aldose reductase (AKR1B1) that requires nicotinamide adenine dinucleotide phosphate hydrogen (NADPH), and sorbitol is oxidized to fructose by sorbitol dehydrogenase (SORD) yielding nicotinamide adenine dinucleotide hydrogen (NADH) from nicotinamide adenine dinucleotide (NAD). The conversion of glucose to fructose may enhance glycolysis with conversion of fructose to fructose-1-PO_4_ (F1P) by ketohexokinase (KHK) occurring mainly in the liver [16, 17]. KHK is also frequently expressed in tumors [18–20] that also produce fructose 1-P endogenously from fructose (a product of glucose). This reaction bypasses the rate-limiting step in glycolysis at the level of phosphofructokinase (PFK), which is inhibited by high levels of ATP, citrate and low pH. Naked mole rats also increase the production of fructose and F1P to increase their survival under low oxygen levels and even anoxia because fructose supports glycolysis and generation of ATP and substrates for other major metabolic pathways [21]. Low concentrations of oxygen inhibit oxidative phosphorylation that lowers pH and inhibits PFK. Thus, metabolism of fructose to F1P by KHK bypasses the PFK feedback inhibition by low pH, whereas glaceraldehyde-3-PO_4_ (GAP) and dihydroxyacetone-PO_4_ (DHAP) enter glycolysis downstream of PFK.

Most tumors develop initially in a hypoxic and acidic environment [22]. A direct link between polyol pathway activity and cancer was reported by Schwab et al. [23] who found a strong correlation between expression of AKR1B1and epithelial-to-mesenchymal transition (EMT) in patients with lung cancer and in an EMT-mediated colon cancer mouse model. They also found that AKR1B1 knockdown decreased EMT in cancers. Knockdown of expression of SORD also suppressed EMT in the cancer cell line. Schwab and colleagues [23] reported that glucose metabolism via the polyol pathway controls EMT via transforming growth factor beta (TGFB) autocrine stimulation and that expression of TGFB decreased following knockdown of AKR1B1 and SORD while EMT markers were rescued by TGFB [23]. There is also a link between the polyol pathway, particularly AKR1B1 overexpression, and development of breast, ovarian, cervical, and rectal cancers [24]. For colorectal cancer cells, AKR1B1 affects cellular proliferation and cell cycle progression, cell motility and expression of nuclear factor kappa-light-chain-enhancer of activated B cells (NFKB) resulting in a poor prognosis for colorectal cancer patients [25].

There is considerable evidence for endogenous production of fructose via the polyol pathway in cancer cells and phosphorylation of fructose to F1P by KHK in tumors of humans. This pathway for glycolysis is clearly advantageous as it bypasses PFK regulation to allow endogenously produced fructose to be used to produce F1P and substrates from the glycolytic pathway to support of cancer cells in a low oxygen, low pH environment that include the hexosamine biosynthesis pathway [26], the pentose cycle (PC) [27], *de novo* lipogenesis [28], one-carbon metabolism [29], and the TCA cycle [29].

## The polyol pathway, fructose synthesis, and pregnancy

The polyol pathway involves two steps for conversion of glucose to fructose [13–15] as reviewed in *Fructose Metabolism and Cancer*. But the first report of fructose in tissues of conceptuses was in 1855 by Bernard [30]. Between that time and 1956, numerous reports confirmed the presence or absence of fructose in blood, amniotic fluid and allantoic fluid of ungulates and other species (see Goodwin [31]). Due to confusion about the presence or absence of fructose in fetal blood among species, Goodwin [31] measured fructose in blood from ungulates (ox, goat, horse, pig), cetaceans (fin, humpback and blue whales), and nonungulates (guinea pig, rabbit, rat, dog, cat, ferret) and cited work indicating the absence of fructose in blood from newborn human babies. Thus, he divided common mammals into two groups: those with detectable fructose (ungulates and cetaceans) and those with very low or undetectable fructose in fetal blood (nonungulates and humans). It was also noted that samples of fetal blood and amniotic fluids from ungulates and cetaceans had much greater concentrations of fructose than glucose.

There are species with invasive implantation and either hemochorial (humans, monkeys and rodents) or endotheliochorial (carnivores) placentae with close apposition between maternal and blood and fetal blood for efficient transport of glucose. Species with those types of placentae have little or no fructose in fetal blood or fetal fluids. However, species with noninvasive implantation have either epitheliochorial placentae (pigs, horses, cetaceans) or syndesmochorial placentae (cows, sheep, goats) with five or six layers of cells between maternal and fetal blood. Those species have fructogenic placenta with fructose being the most abundant hexose sugar in blood and fetal fluids of those species. Fructose is present in fluid of the extra-embryonic coelom of human conceptuses in early gestation [32], but it is a minor sugar compared with glucose. Fructose is also a minor sugar in fetal blood and fetal fluids of dogs, cats, guinea pigs, rabbits, rats, and ferrets [31].

Studies of pregnant ewes [see [6, 33, 34]] revealed that: 1) injection of glucose into ewes to make them hyperglycemic resulted in a rapid increase in fructose in fetal blood; 2) injection of glucose into the fetal vasculature increased glucose in maternal blood and the fetus experienced hyperfructosemia indicating that glucose, but not fructose, could be transported from the fetal-placental vasculature to maternal blood; 3) the placenta is responsible for converting glucose to fructose; and 4) the flux of glucose from maternal blood to fetal blood may reach 70 mg/min in hyperglycemic ewes. Radiolabeled glucose was also shown to be converted to radiolabeled fructose by placentae of pigs. One may speculate about four main points. First, in species with hemochorial and endotheliochorial placenta, the chorion is in very close proximity to maternal blood for highly efficient exchange of glucose and other nutrients, as well as gases. The number of tissue layers separating maternal blood and chorion is three in species with hemochorial placenta, four in species with endotheliochorial placenta, but six in species with syndesmochorial and epitheliochorial placenta. Second, species with hemochorial placenta do not have an allantois as it regresses, like the yolk sac, early in gestation. For carnivores with endotheliochorial placenta, there is an allantoic sac, but it is not well developed. However, for ungulates, a well-developed allantois is characteristic of the placenta. The allantois forms a sac that is a reservoir of water and nutrients (e.g., fructose and glutamine) in storage and available to be recycled into the fetal-placental circulation. Third, the rate of uterine blood flow to support a human pregnancy is estimated to be 0.5 L per minute [35] compared to 2.5 L per minute for pigs [36], 5.95 L per minute for cows [37] and 2 L per minute for sheep during late gestation. The high rates of uterine blood flow for pregnant ungulates are critical for delivery and exchange of sufficient nutrients and gases to support fetal growth and development. Fourth, the placentae of ungulates convert glucose to fructose that is sequestered within the pregnant uterus and unavailable to maternal tissues. Rather, it is metabolized via glycolysis after being phosphorylated at carbon number 1 to F1P. This fructolysis pathway is not inhibited by low pH, citrate or ATP.

**FIGURE 2:**
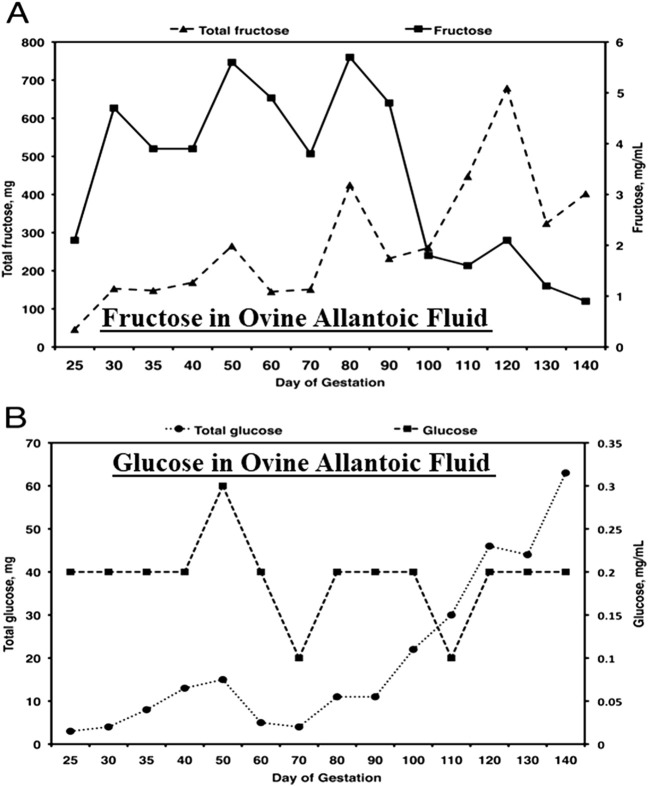
Fructose **(A)**, the most abundant hexose sugar in fetal fluids and fetal blood of ungulates such as sheep, is present at concentrations from 4 to 5 mg between Days 30 and 90 of gestation, the period of rapid placental development, as compared to concentrations of glucose **(B)** of about 0.2 mg/mL. Thus, the concentrations of fructose are some 20- to 30-fold greater than for glucose and, as reported, a unique feature of fructose-1-PO4 it its metabolism to yield ATP, as well as substrates for the pentose cycle, serine for one-carbon metabolism and the hexosamine biosynthesis pathway.

Fructose in conceptuses of sheep and pigs has been ignored by fetal physiologist using those animal models in biomedical research although fructose is 15- to 30-fold greater than glucose in allantoic fluid of sheep (see Figure 2), as well as blood of ovine fetuses (data not shown). A review by Battaglia and Meschia [38] on fetal and placental metabolism does not mention fructose. Fructose was considered a waste product since it was not metabolized via the classical glycolytic pathway [38–42] even though it is 11- to 33-times more abundant than glucose in blood and allantoic fluid of fetal lambs [33]. The literature indicates the following: 1) fructose can be used for synthesis of nucleic acids and generation of reducing equivalents in the form of NADPH in fetal pigs [43] and in HeLa cells [44]; 2) neither fructose nor glucose is metabolized via the PC by the ovine placenta [45]; 3) fructose and glucose are equivalent substrates for the synthesis of neutral lipids and phospholipids in heart, liver, kidney, brain, and adipose tissue of fetal lambs [46]; 4) activities of glucose-6-phosphate (G6P) dehydrogenase, malic enzyme, and acetyl-CoA carboxylase in liver are stimulated by glucose to increase lipogenesis [47]; and 5) fructose enters adipocytes by both insulin-independent and insulin-insensitive mechanisms [48]. These lines of evidence indicated that both glucose and fructose are metabolic substrates utilized by mammalian conceptuses.

## Unique attributes of fructose as a metabolic substrate supporting growth and development of ungulate and cetacean conceptuses

Fructose is 15- to 30-fold greater in abundance than glucose in fetal fluids and plasma throughout pregnancy in sheep, for example, highlighting the facts that ungulate placentae are fructogenic and that fructose, but not glucose, is sequestered within fetal blood and fetal fluids [[49–51]; Figure 2]. The following are key findings from research on metabolism of fructose in ungulates. First, cell-specific, and temporal changes in expression of enzymes involved in the metabolism of glucose and fructose in ovine and porcine conceptuses throughout pregnancy are well characterized [6, 34, 52–59]. Second, cell-specific and temporal expression of KHK isoforms for conversion of fructose to F1P provide a substrate for metabolism via the fructolysis pathway that bypasses the regulatory step at PFK in glycolysis for unimpeded production of key substrates for the PC (G6P) [54], hexosamine biosynthesis (F6P) [6, 59], one-carbon metabolism and serine synthesis (3-phosphoglycerate) [53], and TCA cycle (pyruvate) [54]. Third, the glycolytic pathway generates abundant lactate that is not likely used for gluconeogenesis by ovine conceptuses as they do not express the required enzymes for gluconeogenesis; glucose-6-phosphatase (G6Pase) or phosphoenolpyruvate carboxykinase (*PCK*) [54]. Fourth, ovine conceptuses do express aldolase, an enzyme that converts glyceraldehyde-3-phosphate (GAP) and dihydroxyacetone phosphate to F-1,6-P. The latter is dephosphorylated to F6P, which is converted to G6P via phosphoglucoisomerase (PGI) for metabolism via the PC and glycogen synthesis [54]. Fifth, there is expression of O-linked N-acetylglucosaminyltransferase (*OGT)* mRNA in placentomes of sheep throughout gestation and OGT glycosylates and activates proteins such as AKT (protein kinase B) to inhibit silencing of TSC2 (tuberous sclerosis complex 2) and activate mechanistic target of rapamycin (mTOR) that stimulates expression of mRNAs and proteins involved in cell proliferation and migration [59]. Sixth, F6P and glutamine are required for synthesis of UDP-GlcNAc (UDP-N-acetyl-D-glucosamine) that uses OGT to activate the mTOR pathway in ovine [59] and porcine [6] conceptuses by transferring a carbohydrate moiety to a serine or a threonine residue [59]. Like fructose, glutamine is also unusually abundant in conceptuses of ungulates. For example, concentrations of glutamine in ovine allantoic fluid are approximately 25 mM at Day 60 of gestation [60].

### Fructose synthesis by sheep and pig conceptuses

**FIGURE 3:**
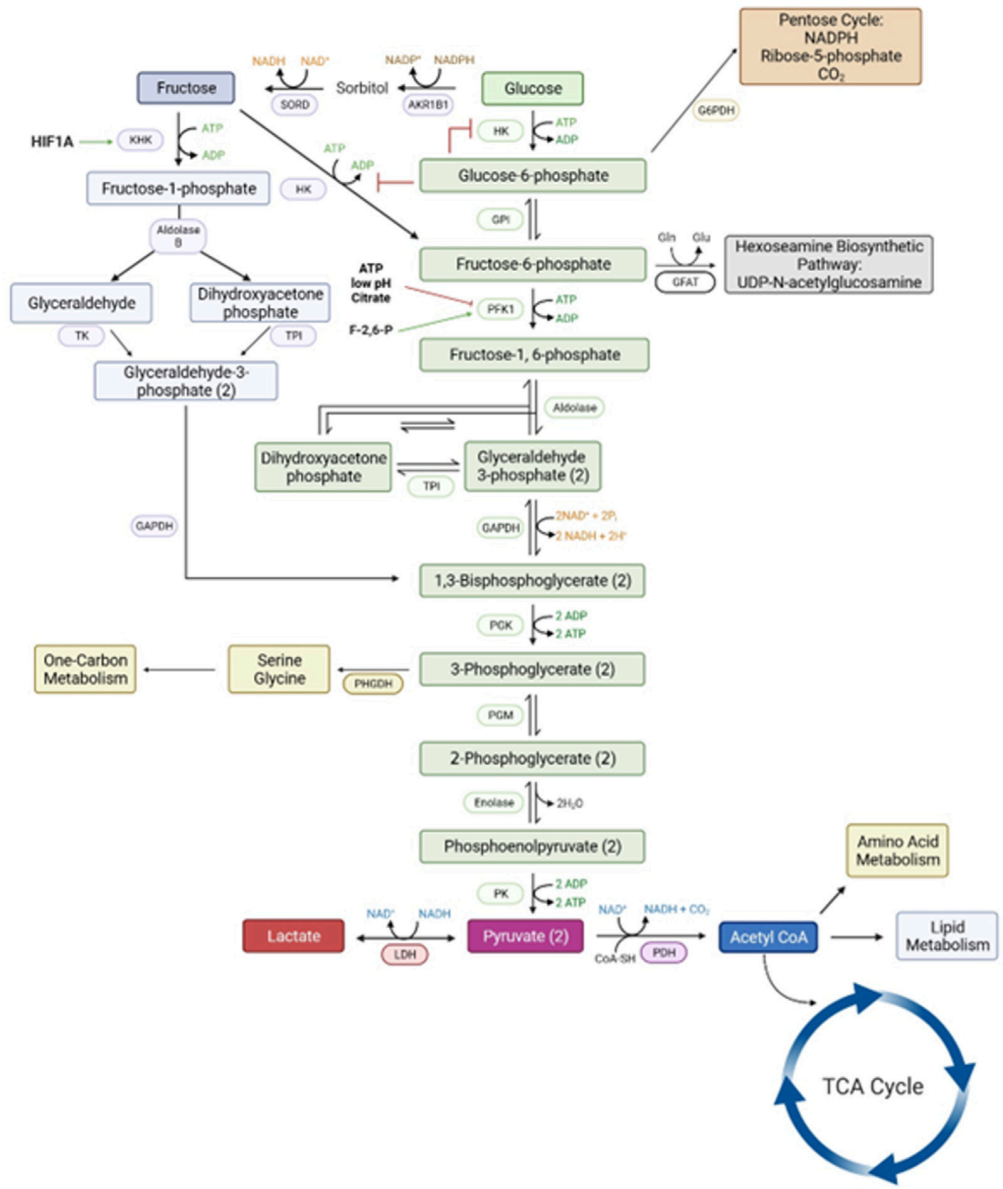
The metabolic pathways for a metabolism of glucose via the classical hexosamine pathway has a rate-limiting step at phosphofructose kinase (PFK1) that is inhibited by ATP, low pH and citrate. However, the conversion of glucose to fructose and fructose to fructose-1-PO4 by ketohexokinase allows for an uninhibited pathway for generation of substrates for all major metabolic pathways required for growth and development of the conceptus. Please see the list of abbreviations for each of enzymes noted in this figure [6, 59, 61].

The period of conceptus development between fertilization and implantation in mammalian species is critical for setting the stage for placental and fetal development. The trophectoderm and endoderm of pre-implantation ovine and porcine conceptuses undergo rapid elongation that involves proliferation, migration, and cytoskeletal modifications of trophectoderm cells. These complex events occur in a low oxygen intrauterine environment supported by nutrients and gases either transported or secreted into the uterine lumen. The conceptus utilizes glucose provided by the mother to initiate metabolic pathways that provide energy and substrates for other metabolic pathways. In ugulates, most available glucose is converted to fructose via the polyol pathway (see Figure 3). As noted previously, even subterranean rodents living in a low oxygen environment, switch to fructose-driven metabolism in very low oxygen environments [21] and cancer cells also produce and metabolize fructose as an adaptation to low oxygen environments [61].

The cell-specific and temporal expression of enzymes required for synthesis and metabolism of fructose in sheep and pig conceptuses include those for the polyol pathway (*SORD* and *AKR1B1*) and glucose and fructose metabolism (*HK1*, *HK2*, *G6PD*, *OGT*, KHK, and *FBP)*, but not those required for gluconeogenesis (*G6Pase* or *PCK*) [52, 57]. Ovine placentomes also express mRNAs for *SORD*, *AKR1B1*, *HK1*, and *OGT,* as well as two isoforms of ketohexokinase, (KHKA and KHKC). The KHKA and KHKC isoforms are expressed in ovine conceptuses from Day 16 of pregnancy and placentomes throughout pregnancy in a cell specific manner. KHKA is most abundant in trophectoderm and cotyledons of placentomes, while KHKC is more abundant in endoderm of Day 16 conceptuses and chorionic epithelium of placentomes. Expression of *KHK* mRNAs in placentomes is greatest at Day 30 of pregnancy in sheep, but not different among days later in gestation.

Fetal fluids and uterine flushings from pigs contain higher concentrations of fructose than glucose, but fructose is not detected in maternal blood. As noted for sheep, fructose is derived from glucose via enzymes of the polyol pathway, AKR1B1 and SORD, transported across cell membranes by solute carriers SLC2A5 and SLC2A8, and converted to F1P by KHK [58]. For pigs, progesterone up-regulates SLC2A8 expression in uterine luminal (LE) and glandular (GE) epithelia during the peri-implantation period of pregnancy and in chorion and blood vessels after Day 30 of gestation. Progesterone up-regulates SLC2A5 mRNA in uterine LE and GE after implantation, and the chorion expresses SLC2A5 between Days 30 and 85 of gestation in pigs. AKR1B1 and SORD are expressed by uterine LE during the peri-implantation period, but expression switches to chorion by Day 20 and is maintained through at least Day 85 of pregnancy. Expression of AKR1B1 mRNA in the uterus is downregulated by estrogen. KHK is expressed by trophectoderm/chorion throughout gestation. Enzymes for conversion of glucose to fructose and for fructose transport are present at the uterine-placental interface of pigs [57, 58]. The shift in expression from uterine LE to chorion during pregnancy suggests that free-floating conceptuses of ungulates are supported initially by fructose synthesized by the uterus, but after implantation, the chorion becomes self-sufficient for fructose synthesis and transport in pigs and sheep [57].

### Fructose and the hexosamine biosynthesis pathway

A porcine trophectoderm cell line (pTr1) was used to demonstrate that both fructose and glucose increase cell proliferation by increasing phosphorylated-RPS6K, -EIF4EBP1, and -RPS6 over basal levels within 30 min; an effect sustained for 120 min [6]. Those effects were inhibited by specific inhibitors of RPS6K, EIF4EBP1, PI3K and MTOR. In the same study, the biosynthesis of hyaluronic acid from glucose and fructose was investigated [59]. Inhibition of glutamine-fructose-6-phosphate transaminase 1 (GFPT1) by azaserine (an inhibitor of GFPT1) and GFPT1 siRNA, revealed that MTOR-RPS6K and MTOR-EIF4EBP1 signaling in response to fructose is mediated via GFPT1 activation and the hexosamine pathway. Further, both glucose and fructose are utilized for the production of hyaluronic acid via GFPT1 and the hexosamine biosynthesis pathway. Hyaluronic acid, also known as hyaluronan, is an anionic nonsulfated glycosaminoglycan found as a major component of the extracellular matrix in many tissues, including cancers and in placentae it is known as Wharton’s Jelly [62–64]. During placental development hyaluronic acid is an abundant extracellular matrix component responsible for tissue hydration and hydrodynamics, cell migration and proliferation, cell adhesion, supporting blood vessels and enhancing angiogenesis, and it serves as a niche for stem cells [65–67].

Partial degradation products of sodium hyaluronic acid, specifically fragments of 4 and 25 disaccharides in length, elicit angiogenesis in chick chorioallantoic membranes, whereas the intact high molecular weight hyaluronic acid does not induce angiogenesis [68]. Vallet et al. [69] reported that hyaluronic acid in the placenta of pigs increases between Days 25 and 45 of gestation and remains high throughout gestation, while expression of hyaluronoglucosaminidases 1 and 2 also increased with advancing gestation in pigs. Thus, through its role as a substrate for the hexosamine biosynthesis pathway for synthesis of hyaluronic acid, fructose influences angiogenesis and development of microscopic folds of the placenta [69].

Both glucose and fructose can be metabolized to fructose-6-P, as noted previously. Glutamine:fructose-6-P transaminase (GFPT1) then converts glutamine and fructose-6-P into glucosamine-6-P, which is a common substrate for the formation of all aminosugars and glycoproteins in animal cells. Fructose and glucose, in cooperation with glutamine, may affect proliferative behavior of conceptus trophectoderm/chorion via activation of the Akt-TSC2-MTOR signaling cascade. The phosphorylation for activation of this cascade is mediated by O-GlcNAcylation from UDP-N-acetylglucosamine, a primary product of the hexosamine biosynthesis pathway. Key roles of fructose in cellular functions equivalent to those of glucose are to activate integrated cell signaling pathways affecting proliferation of trophectoderm cells through metabolism via the nonoxidative hexosamine biosynthesis pathway to produce GlcN-6-P by GFPT1, increase O-GlcNAcylation of cellular proteins/enzymes by OGT, and increase phosphorylation of the Akt-TSC2-MTOR cell signaling cascade [59]. Fructose and glucose stimulate proliferation of ovine trophectoderm cells at 4 mM, but concentrations of fructose of 11.1–33.3 mM may have much greater effects than glucose at concentrations of only 0.6–1.1 mM. The hexosamine biosynthesis pathway yields UDP-GlcNAc for cytosolic and Golgi-mediated O- linked glycosylation (O-GlcNAcylation) of proteins and glycosylphosphatidylinositol anchors proteins to the outer plasma membrane. The O-GlcNAcylation is the process whereby β-D-N-acetylglucosamine is added to serine or threonine residues of proteins, and OGT is required for this process affecting proliferation and adhesion of oTr1 cells as well as activation of the Akt-TSC2-MTOR signaling cascade. Knockdown of translation of OGT mRNA in oTr1 cells using a morpholino antisense oligonucleotide (MAO) and inhibiting OGT by alloxan both significantly decreased fructose-induced total protein O-GlcNAcylation and cell proliferation and fructose-induced phosphorylation of MTOR, P70S6K, and 4EBP1. In addition, the synthesis of glucosamine-6-P is highly active in tumor cells, which are known to extensively use glutamine for OGT and mTOR activation, as well as cell growth and development (Figure 5) (Akella et al. 2019). Thus, there is evidence for fructose-induced cell proliferation and activation of MTOR cell signaling being regulated by O-GlcNAcylation.

### Fructose metabolism supports the PC and TCA cycle

The culture of ovine conceptus homogenates with ^14^C-labeled glucose and/or ^14^C-labeled fructose under oxygenated and low oxygen conditions was conducted to assess contributions of glucose and fructose to the PC, TCA cycle, synthesis of glycoproteins and the synthesis of lipids [54]. Both glucose and fructose contributed carbons to each of those pathways, except for lipid synthesis, and both hexose sugars were metabolized to pyruvate and lactate, with lactate being the primary product of glycolysis under both oxygenated and low oxygen conditions. The ovine conceptus tissue preferentially oxidized glucose over fructose and incorporation of fructose and glucose at 4 mM each into the PC by Day 16 conceptus homogenates was similar in the presence or absence of glucose, but incorporation of glucose into the PC was enhanced by the presence of fructose. The incorporation of fructose into the PC in the absence of glucose was greater under oxygenated conditions, and incorporation of glucose into the PC under low oxygen conditions was greater in the presence of fructose. These results indicate that both glucose and fructose are important metabolic substrates for metabolism via the PC, TCA cycle, and synthesis of glycoproteins [see [34, 54]].

### Fructose metabolism supports one-carbon metabolism

Conceptuses of sheep and pigs, for example, undergo incredibly rapid increases in elongation during the peri-implantation period of pregnancy requiring rapid increases in proliferation and migration of trophectoderm that requires equally rapid increases in metabolic reactions that generate nucleic acids for synthesis of DNA and RNA, but also for other vital pathways such as those that generate ATP, reducing agents, or intermediates for subsequent reactions [55, 70, 71]. However, for survival during the peri-implantation period of pregnancy, the conceptus is entirely reliant upon the histotroph secreted and/or transported by uterine epithelia into the uterine lumen including glucose that is rapidly metabolized to fructose. A key amino acid for 1C metabolism is serine, the second most abundant amino acid (following glycine) in uterine flushings from pregnant ewes that increases 6.2-fold between Days 10 and 16 of gestation [60]. Also, with advancing stages of gestation, serine is the most abundant amino acid in fetal blood and allantoic fluid of sheep [72]. Serine can also be synthesized from glucose and/or fructose via the serinogenesis pathway in which 3-phosphoglycerate (3PG, a glycolytic intermediate) is converted to serine by the sequential enzymatic conversions of phosphoglycerate dehydrogenase (PHGDH), phosphoserine aminotransferase 1 (PSAT1), and phosphoserine phosphatase (PSPH) [73]. The conversion of both sugars into serine requires glutamate, a metabolite of glutamine via phosphate-activated glutaminase. The 1C metabolism pathway uses serine as a substrate for transferring 1C units (i.e., methyl groups) linking together the folate cycle that also provides 1C units and the methionine cycle that recycles components of the folate cycle in healthy tissues [74] and cancerous tissue [75]. Ultimately, the production of 1C units via the folate cycle is for the production of formate required for the synthesis of adenine, guanidine, and thymidine nucleotides [76]. Also, 1C metabolism is important for generation of S-adenosylmethionine (SAM) through the methionine cycle and SAM is required for methylation of nucleic acids and proteins for epigenetic modifications [77]. (NADPH generated via 1C metabolism also impacts mitochondrial redox control, particularly under conditions of low oxygen [77].

One-carbon metabolism is critical for metabolism in cancer cells [78, 79]. As cancer cells are highly proliferative under low oxygen conditions like developing conceptuses of ungulates during the peri-implantation period of pregnancy, cancer cells maintain a proliferative state while oxygen deprived by upregulating serine catabolism and 1C metabolism [7, 78]. Therefore, conceptuses of livestock species undergoing extensive cellular proliferation and rapid elongation likely utilize similar metabolic pathways but must rely on extracellular nutrients secreted and/or transported from maternal blood into the uterine lumen, such as glucose, fructose, and serine for 1C metabolism for production of formate during the peri-implantation period of pregnancy.

An experiment was conducted to demonstrate that in addition to free serine available to the conceptus *in utero*, glucose and fructose can generate serine via the serinogenesis pathway in ovine conceptuses for production of formate required for synthesis of purines and thymidine for nucleic acid synthesis [53]. Ovine conceptuses from Day 17 of gestation were cultured in medium containing either: 1) 4 mM D-glucose + 2 mM [U-^13^C]serine; 2) 6 mM glycine + 4 mM D-glucose + 2 mM [U-^13^C]serine; 3) 4 mM D-fructose + 2 mM [U-^13^C]serine; 4) 6 mM glycine + 4 mM D-fructose + 2 mM [U-^13^C]serine; 5) 4 mM D-glucose + 4 mM D-fructose + 2 mM [U-^13^C]serine; or 6) 6 mM glycine + 4 mM D-glucose + 4 mM D-fructose + 2 mM [U-^13^C]serine to determine production of formate. The ovine conceptuses produced both ^13^C- and ^12^C-formate, indicating that the [U-^13^C] serine, glucose, and fructose were utilized to generate formate, respectively. Greater amounts of ^12^C-formate than ^13^C-formate were produced, indicating that ovine conceptuses utilized more glucose and fructose than serine to produce formate. These results were the first to demonstrate that both 1C metabolism and serinogenesis are active metabolic pathways in ovine conceptuses during the peri-implantation period of pregnancy, and that both glucose and fructose are substrates for generating formate required for synthesis of nucleotides and SAM in rapidly proliferating trophectoderm cells.

### Fructose and production of lactate via glycolysis

**FIGURE 4:**
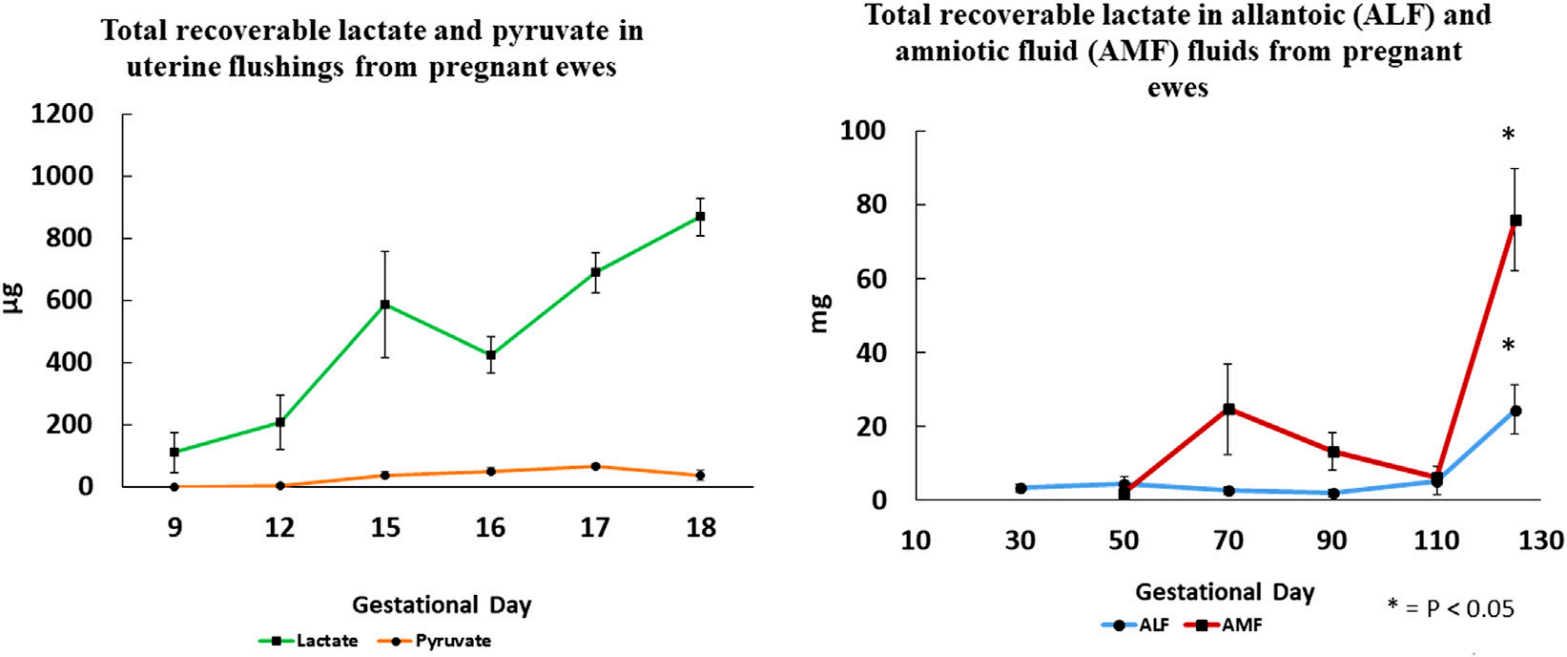
Total recoverable lactate in uterine flushings increases significantly between Days 9 and 18 of gestation, while pyruvate is barely detactable suggesting that lactate dehydrogenase A that converts pyruvate to lactate is the most active isoform. Changes in total recoverable lactate in amniotic and allantoic fluids are also shown with respect to day of gestation and fetal fluid compartment.

Lactate, an abundant molecule in fetal fluids and blood of mammalian species is, like fructose, overlooked as a metabolic waste product generated during pregnancy. The metabolism of both glucose and fructose by ovine conceptuses generates significant amounts of lactate. Moses et al. [80] characterized lactate production by ovine conceptuses throughout gestation, as well as expression of mRNAs and proteins involved in lactate metabolism. Lactate in the uterine lumen of sheep increases during the preimplantation period of pregnancy and is significantly more abundant than pyruvate (see Figure 4). Also, concentrations and total amounts of lactate in allantoic and amniotic fluids increase with advancing days of gestation and most abundant on Day 125 of pregnancy. Lactate dehydrogenase (LDH) subunit A (converts pyruvate to lactate) and subunit B (converts lactate to pyruvate) are expressed by ovine conceptuses throughout gestation. Lactate is transported via monocarboxylic acid transporters (MCT) 1 and 4, both of which are expressed by the conceptus throughout gestation. Additionally, the inter-placentomal chorioallantois from Day 126 expresses MCT1 and MCT4 for transport of lactate. Hydrocarboxylic acid receptor 1 (HCAR1) is the receptor for lactate localized to the uterine LE and sGE, as well as conceptus trophectoderm of pregnant ewes throughout gestation [80].

## Lactate receptors

The G-protein-coupled receptors for lactate, GPR81 (hydroxycarboxylic receptor 1), GPR109A (hydroxycarboxylic receptor 2) and GPR109B (hydroxycarboxylic receptor 3) share high sequence homology and are designated HCAR1, HCAR2 and HCAR3 [81, 82]. HCAR1 is activated by lactate whereas HCAR2 is activated by the ketone body 3-hydroxy-butyric acid, and HCAR3 is activated by 3-hydoxy-octanoic acid (an intermediate in β-oxidation of octanoate). Lactate binding to HCAR1 stimulates GTPγS-binding with an EC50 of 1.3 mM. However, HCAR1 cloned from tissues of mice, rats, dogs, pigs, cows, monkeys, and Zebra fish responds to physiological concentrations of lactate at 0.5–2.0 mM, as well as 10–20 mM lactate. HCAR1 is expressed in human pituitary, adipocytes, and brown adipose tissue, as well as uterine epithelia and conceptus trophectoderm of sheep [see [80]]. In tumor cells, lactate regulates expression of HCAR1 mRNA via STAT3 (signal transducer and activator of transcription factor 3) cell signaling. In non-immune cells, lactate-HCAR1 cell signaling activates protein kinase A (PKA) and ERK (extracellular signal-related kinases) pathways, and plasmacytoid dendric cells are induced to express interferon alpha (IFNA) in response to calcium mobilization and calcium-calmodulin dependent protein kinase II (CaMKII) and calcineurin (CaN) phosphatase [82]. HCAR1 protein is expressed by uterine LE and sGE, as well as trophectoderm, but not GE, myometrium from sheep on Day 17 of pregnancy, as well as all cell types of the uterus on Days 30, 70, 90, 110, and 125 of gestation [80].

### Lactate as a cell signaling molecule

Expression of hypoxia inducible factor 1 (HIF1A) may occur under both hypoxic and normoxic environments; however, if HIF1A proline residues are hydroxylated by prolyl-4-hydroxylase (PHD) HIF1A is ubiquitinated by the von Hippel-Landeu protein and degraded. But, in the presence of lactate, PHD is inhibited and HIF1A is not subject to degradation [see [83]]. Tumor-derived lactate activates endothelial cells and stimulates angiogenesis through both HIF1A-dependent and HIF1A-independent pathways [84, 85]. In the HIF1A-dependent pathway, MCT1 transports lactate into endothelial cells to inactivate PHDs and stabilize HIF1A that then induces expression of vascular endothelial growth factor (VEGF) to promote angiogenesis in tumor cells under normoxic conditions [86]. Lactate may also induce angiogenesis via a HIF1A-independent mechanism by binding directly to N-Myc downstream-regulated protein (NDRG3) and preventing HIF1A degradation by PHD [87]. NDRG3 promotes angiogenesis under conditions of low oxygen and high concentrations of lactate by binding to c-Raf and activating Raf-ERK signaling in tumor cells to sustain HIF1A activity required for tumorigenesis. Constitutive HIF1A is detectable in non-hypoxic cancer cell lines in response to lactate and pyruvate as evidenced by the accumulation of HIF1A protein in many cancer cell lines due primarily to lactate that prevents degradation of HIF1A [88].

## Lactate, HCAR1, and pregnancy

HIF1A is important for the establishment and maintenance of pregnancy in mammals as conceptuses develop in a low oxygen environment and respond to changes in oxygen tension, hormones, and other molecules. For example, expression of HIF1A is upregulated by progesterone in the uterus [89, 90], while HIF2A is upregulated by estrogen [90]. In sheep, HIF1A mRNA is induced by progesterone in the endometrium and HIF2A is upregulated in response to progesterone and IFNT [89]. Lactate is a ligand for HCAR1 in mammary tumors and is designated an orphan G-protein coupled receptor [91]. Lactate interactions with HCAR1 in cancer cells [92] promote angiogenesis [91], tumor growth [93], and chemoresistance [94], and proliferation and migration of normal cells [92]. Lactate produced via glycolysis is used potentially as: 1) an energy source for mitochondrial respiration; 2) gluconeogenic precursor; and 3) cell signaling molecule at physiological concentrations of lactate from 0.5 to 20 mM and when the lactate/pyruvate ratio ranges from 10 to greater than 500 mM under conditions such as vigorous exercise and stress [81]. Lactate acting via HCAR1 in adipocytes inhibits lipolysis by decreasing mitochondrial fatty acid uptake via malonyl-CoA and carnitine palmitoyltransferase I in muscle [84].

The high lactate and low pH environment in the uterine lumen during early pregnancy is created by lactate produced by blastocysts in mice [see [95–97]]. Lactate and low pH increases expression of mRNAs for VEGFA, HCAR1, SLC2A4 (also known as glucose transporter member 4), transcription factor p65 (RELA), MCT1 and snail (SNAI1) involved in epithelial to mesenchymal cell transition in Ishikawa cells. Lactic acid also increases migration of decidualized stromal cells in uteri of mice without changing the extent of decidualization. Further, human umbilical vein endothelial cells (HUVEC) form tubes when treated with 5 mM lactic acid as evidence of an angiogenic effect of lactic acid. Garner [95] reported that mammalian blastocysts use aerobic glycolysis as do cancer cells to create a microenvironment in which the pH is low to increase angiogenesis, vascular permeability, tissue disaggregation through breakdown of the extracellular matrix associated with increases in expression of matrix metalloproteinases 1 and 2 (MMP1 and 2) from blastocysts and MMP9, transforming growth factors beta 1 and 2 (TGFB1, TGFB2), cathepsin B and hyaluronic acid. The increase in hyaluronic acid is suggested to increase hydration of the endometrium due to facilitate implantation. Also, there was a decrease in expression of tissue inhibitors of metalloproteinases (TIMPs) and an increase in NFKB in that study [95]. Lactate also increased Treg cells, conversion of macrophages from M1 (inflammatory) to M2 (anti-inflammatory) phenotypes, and expression of VEGF in macrophages. Gardner [95] suggests that post-implantation conceptuses become more dependent on glycolysis to produce lactate and maintain a low pH environment that mimics hypoxia.

Comline and Silver [98] reported concentrations (mg/100 mL) of lactic acid in fetal umbilical vein blood at 9, 5 and 1 day prepartum to be 16.7, 16.8, and 19.1, respectively, as compared to values in blood from the maternal uterine vein of 11.2, 9.8 and 9.8. In comparison, concentrations (mg/100 mL) of fructose in fetal umbilical vein blood at 9, 5, and 1 day prepartum were 74.3, 77.8, and 67.7, respectively, but not detectable in maternal blood samples. The concentrations of glucose in umbilical vein blood were 13.3, 12.7, and 19.1 mg/100 mL at 9, 5 and 1 day prepartum, respectively as compared to 57.6, 58.0, and 68.0 mg/100 mL in maternal uterine vein blood. Thus, concentrations of fructose in fetal umbilical vein blood were 5- to 6-fold greater than those for glucose in that study [98].

## Summary

The literature documents that the intrauterine environment for mammalian conceptuses is hypoxic relative to normal air [95, 99–102] and that is also true for developing tumors [61]. Accordingly, the polyol pathway is active in conceptuses of ungulates such as pigs [57] and sheep [54] and even humans [32]. The polyol pathway generates fructose and the metabolism of fructose via frutolysis is sustained for conceptuses of pigs [57] and sheep [33] throughout gestation, but not for human conceptuses [32]. We speculate that species with invasive implantation and hemochorial or hemoendothelial placenta with fewer layers of tissue separating maternal and fetal blood do not require high rates of blood flow to the uterus or a well-developed allantois to serve as a reservoir of nutrients. The various species of ungulates have superficial implantation of the blastocyst/conceptus and high rates of uterine blood flow to ensure a sustained delivery of high amounts of nutrients [concentration of nutrient X uterine blood flow] for transfer to the fetal-placental vasculature, as well as a well-developed allantois in which nutrients not utilized immediately can accumulate and be recycled to ensure that the conceptus is well nourished.

**FIGURE 5:**
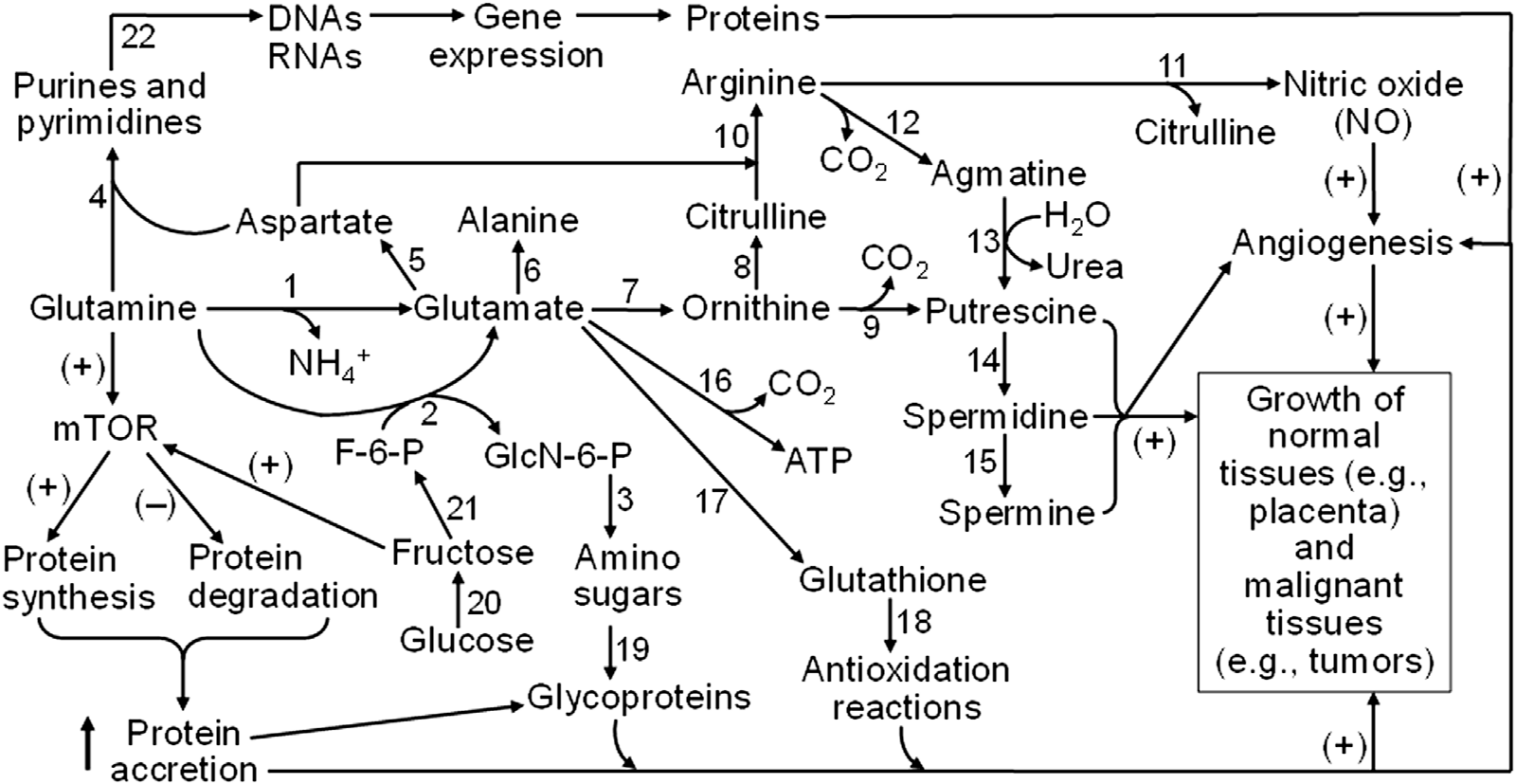
Mechanisms responsible for glutamine and fructose metabolism to stimulate the growth of both normal and malignant tissues in humans and animals. Glutamine is degraded via multiple pathways to generate glutamate, aspartate, alanine, ornithine, citrulline, arginine, glucosamine, and CO_2_, purines, and pyrimidines. Ornithine and arginine are substrates for the synthesis of polyamines (putrescine, spermidine, and spermine), whereas nitric oxide (NO) is formed from arginine oxidation. Glutamate is required for the production of glutathione as the most abundant low-molecular-weight antioxidant. Amino sugars (which deriving the carbohydrate moiety from fructose-6-phosphate, a product of fructose) are required for the generation of glycoproteins as essential components of membranes, cytoplasm, and the extracellular matrix. Purine and pyrimidine nucleotides are precursors of DNAs and RNAs that are necessary for protein synthesis. Furthermore, both glutamine and fructose activate the mTOR cell signaling pathway to stimulate protein synthesis and inhibit protein degradation, leading to protein accretion in cells. NO (a major vasodilator to promote blood flow and nutrient supply), polyamines, protein accretion, and redox balance are crucial for angiogenesis and the growth of both normal and malignant tissues in humans and animals. Abbreviations: F-6-P, fructose-6-phosphate; GlcN-6-P, N-acetylglucosamine-6-phosphate; mTOR, mechanistic target of rapamycin. The enzymes that catalyze the indicated reactions are: (1) phosphate-activated glutaminase; (2) glutamine:fructose-6-phosphate transaminase; (3) glucosamine-phosphate N-acetyltransferase, phosphoacetylglucosamine mutase, UDP-GlcNAc pyrophosphorylase, and UDP-GlcNAc 4-epimerase; (4) a series of enzymes for purine and pyrimidine syntheses; (5) glutamate-oxaloacetate transaminase; (6) glutamate-pyruvate transaminase; (7) pyrroline-5-carboxylate synthetase and ornithine aminotransferase; (8) ornithine carbamoyltransferase; (9) ornithine decarboxylase; (10) argininosuccinate synthase and argininosuccinate lyase; (11) nitric oxide synthase; (12) arginine decarboxylase; (13) agmatinase; (14) spermidine synthase; (15) spermine synthase; (16) a series of enzymes for glutamate oxidation (including glutamate transaminases and glutamate dehydrogenase); (17) γ-glutamyl-cysteine synthetase and glutathione synthetase; (18) glutathione-dependent antioxidative enzymes (including glutathione peroxidase, glutathione *S*-transferase, and thioltransferase); (19) a series of enzymes for incorporation of amino sugars into proteins; (20) aldose reductase and sorbitol dehydrogenase; (21) fructokinase and hexokinase; and (22) a series of enzymes for DNA and RNA syntheses. The sign (+) and (−) denote activation and inhibition, respectively.

Adaptation of the polyol pathway and fructolysis in the placenta has several advantages for ungulates (see Figure 5). First, the trophectoderm/chorioallantois rapidly converts available glucose to fructose that cannot be transferred to the maternal vasculature, it is a sequestered hexose sugar. Second, fructose is phosphorylated at carbon 1 to F1P that is then committed to the fructolysis pathway for further metabolism to substrates that support the pentose cycle, TCA cycle, hexosamine biosynthesis pathway, and one-carbon metabolism. Third, the fructolysis metabolic pathway is not inhibited by low pH, ATP or citrate as is the case for the hexosamine-dependent pathway for glycolysis. Fourth, the fructose in fetal blood is excreted in urine during the first 24–48 h after birth so that does not cause insulin-insensitivity [102] as piglets fail to survive on synthetic diets containing only fructose [103, 104]. Failure of newborn piglets to survive on fructose-based synthetic diets further validates the unique role of fructose and the fructolysis pathway used by fetal-placental tissue of ungulates. Further, it is now becoming apparent that lactate, a product of fructose metabolism, likely acts via its own receptor (HCAR1) to influence implantation and placentation, as well as sustain expression of HIF1A and its downstream genes such as KHK and VEGF [105–107]. On-going and future research will further expand knowledge of the roles of fructose and lactate in fetal-placental development and cancer as they represent rapidly developing tissues that share many metabolic profiles.
